# CDK-Mediated Regulation of Cell Functions via c-Jun Phosphorylation and AP-1 Activation

**DOI:** 10.1371/journal.pone.0019468

**Published:** 2011-04-29

**Authors:** Tony J. Vanden Bush, Gail A. Bishop

**Affiliations:** 1 Department of Microbiology, The University of Iowa, Iowa City, Iowa, United States of America; 2 Department of Internal Medicine, The University of Iowa, Iowa City, Iowa, United States of America; 3 Iowa City Veterans' Affairs Medical Center, Iowa City, Iowa, United States of America; Massachusetts General Hospital, United States of America

## Abstract

Cyclin-dependent kinases (CDKs) and their targets have been primarily associated with regulation of cell-cycle progression. Here we identify c-Jun, a transcription factor involved in the regulation of a broad spectrum of cellular functions, as a newly recognized CDK substrate. Using immune cells from mouse and human, and several complementary *in vitro* and *in vivo* approaches including dominant negative protein expression, pharmacologic inhibitors, kinase assays and CDK4 deficient cells, we demonstrate the ability of CDK4 to phosphorylate c-Jun. Additionally, the activity of AP-1, a ubiquitous transcription factor containing phosphorylated c-Jun as a subunit, was inhibited by abrogating CDK4. Surprisingly, the regulation of c-Jun phosphorylation by CDK4 occurred in non-dividing cells, indicating that this pathway is utilized for cell functions that are independent of proliferation. Our studies identify a new substrate for CDK4 and suggest a mechanism by which CDKs can regulate multiple cellular activation functions, not all of which are directly associated with cell cycle progression. These findings point to additional roles of CDKs in cell signaling and reveal potential implications for therapeutic manipulations of this kinase pathway.

## Introduction

Progression of eukaryotic cells through the cell cycle is controlled by serine/threonine kinases known as Cyclin Dependent Kinases (CDKs). Early studies utilizing cell lines established the dependence of transition from G0/G1 into the S phase upon CDK 4, 6, and 2-controlled checkpoints [1]. However, various CDK-deficient mice are viable, [2], [3], [4], [5] although displaying cell-type specific abnormalities [4], [5], [6], [7]. Thus, while individual CDKs are dispensable for mammalian development, they have cell type-specific functions [7]. These activities include cytoskeletal rearrangement, anti-apoptotic signaling, cell adhesion and cell mobility [8], [9], [10], [11]. Whereas the molecular interactions of CDKs in cell cycle progression are well studied, the mechanisms involved in these additional roles are currently unknown. It is hypothesized that the non-proliferative functions mediated by CDKs involve previously unidentified CDK targets [10].

Stimulation of cells through receptors or via changes in environmental conditions (e.g. heat, salinity, pH) induces activation of the stress activated protein kinases (SAPK), including c-Jun N-terminal Kinase (JNK) [12], [13]. JNK activation mediates direct phosphorylation of its substrate c-Jun [12]–[14]. Upon phosphorylation, c-Jun forms homo or heterodimers with other AP-1 family members to form an active AP-1 transcription complex [14]. AP-1 dimers of distinct composition preferentially enhance transcription of a wide variety of target genes, including other AP-1 family subunits [15]. Thus, the enhanced production of AP-1 subunits increases the complexity and consequences of initial AP-1 activation. Initial JNK and c-Jun activities are therefore extremely important in orchestrating diverse cellular responses. We've previously shown that increased c-Jun phosphorylation does not always correlate with JNK activity in B lymphocytes, suggesting that other kinase(s) can regulate c-Jun, and therefore AP-1, functions [16].

Here we demonstrate that CDK4 directly phosphorylates c-Jun in B lymphocytes and dendritic cells (DC) independently of cell proliferation, regulating AP-1 activity and AP-1-regulated cytokine production. In addition to the discovery of an important new CDK substrate that broadens the role of CDKs in cellular function, these findings have implications for potential therapeutic manipulation of CDK family members [17], [18], [19].

## Results

### The effects of CDK inhibitors on phosphorylation of c-Jun and cyclin D production

Stimulation of B cells through either the innate immune receptor Toll-like receptor (TLR) 7 or the adaptive immune costimulator CD40 activates multiple MAPKs, including JNK [16], [20]. Activated JNK phosphorylates and activates the substrate c-Jun. Active c-Jun then homodimerizes or heterodimerizes with members of the c-Jun, cFos, or ATF families to form the transcription factor AP-1 [15], [21]. However, in B cells stimulated through TLR7 and CD40 – together or individually, the activity of JNK is temporally disconnected from c-Jun phosphorylation with c-Jun phosphorylation persisting in the absence of detectible active JNK [16]. Stimulation through both TLR7 and CD40 results in the most profound separation between JNK activation and c-Jun phosphorylation (16). Therefore, this dual stimulation was used in the present studies.

**Figure 1:**
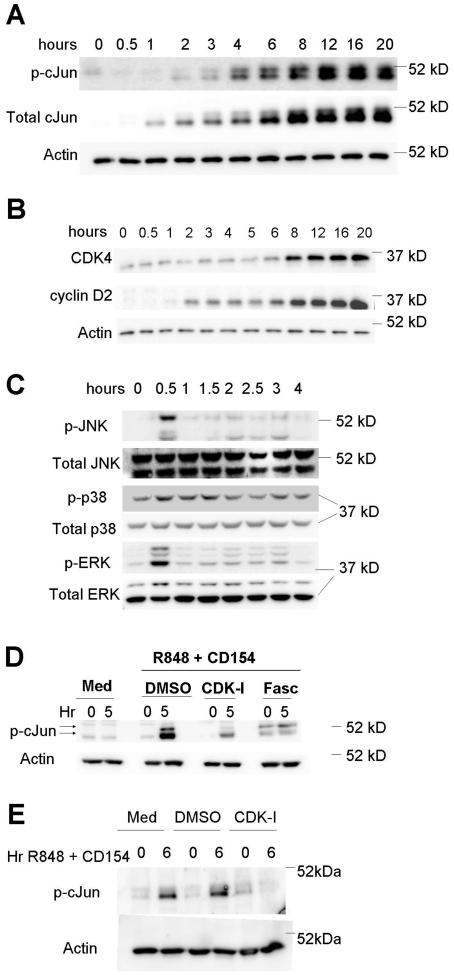
JNK independent cJun phosphorylation. Purified B lymphocytes were stimulated through both CD40 and TLR7 for indicated times. Cells were lysed and analyzed by Western blot for A) phospho–cJun, total -cJun and Actin as a loading control and B) CDK4 and cyclin D2. C) Resting splenic B lymphocyets were stimulated through TLR7 and CD40 (R848 1 ug/ml and CD40L) for the indicated times. Cells were lysed and analyzed for phosphorylated MAP kinases by Western blot. D) Mouse high density splenic B cells or E) human peripheral B cells were stimulated through both CD40 and TLR7 for the designated times in the absence or presence of CDK4 inhibitors SU9516 – CDK-I (10 uM) or Fascaplysin (5 uM) for 5 hours. Cells were then lysed and analyzed for phospho-cJun by Western blot. Results are representative of >3 separate experiments.

While JNK activation peaked and subsided within 60 minutes of dual CD40+TLR7 stimulation, the phosphorylation of c-Jun was first measurable at 30 minutes, continued to increase over 6 hours and remained elevated for up to 20 hours (Fig. 1). Because active c-Jun allows formation of the AP-1 transcription factor, which promotes c-Jun production [15], total c-Jun also increased during this time, requiring the use of actin as a loading control (Fig. 1). The continued increase in p-c-Jun levels hours after JNK activity had diminished suggests that other kinases make important contributions to the sustained phosphorylation of c-Jun, a possibility we wished to investigate. Members of the MAPK/SAPK family such as p38 and ERK were potential candidates as they also phosphorylate c-Jun [22]. However, the kinetics of p38 and ERK activation in response to dual stimulation via CD40 and TLR7 were similar to those of JNK (Fig. 1). These results, together with the relatively large increase in c-Jun phosphorylation seen beyond 60 minutes, suggested that an additional kinase capable of phosphorylating c-Jun was active during early TLR7+CD40 signaling events.

Interactions between the JNK signaling pathway and components of the cell cycle machinery have been reported [19], [23], [24]. Specifically, pharmacologic inhibition of CDKs in neurons affects activity of AP-1 and direct phosphorylation of c-Jun by CDK3 has been observed in response to erythropoietin receptor signaling in epithelial cells [23], [24]. As phosphorylated c-Jun is a subunit of AP-1, we hypothesized that CDKs may be involved in the sustained phosphorylation of c-Jun. Positive regulation of CDK activity depends upon the expression of small polypeptide co-enzymes called cyclins [25]. Early proliferative events dependent upon CDK4 activity, such as the transition of cells from G0/G1 to S phase, are initiated by *de novo* expression of cyclin D family members [26]. Thus, to determine the potential for CDK4 activity during the initial 8 h of stimulation, activated B cells were analyzed for levels of cyclin D2 (the predominant cyclin D family member in B cells) [27], [28]. CDK4 was active in as little as 60 minutes following receptor engagement, as demonstrated by detectable increases in total cellular cyclin D2 (Fig. 1B). While the presence of cyclin D2 indicates CDK4 activity, the role of its activity in c-Jun phosphorylation was still unknown. As an additional test of our hypothesis, the effect of inhibiting CDKs on the phosphorylation of c-Jun was monitored. Each of two structurally distinct CDK inhibitors, SU9516 and Fascaplysin, abrogated the accumulation of phosphorylated c-Jun in stimulated mouse (Fig. 1C) and human B cells (Fig. 1D). Thus, CDKs emerged as potential candidate kinases to regulate c-Jun phosphorylation.

### Requirement for CDK activity in AP-1 activation and IL-6 production

Pharmacologic kinase inhibitors are useful as detectors of potential kinase involvement in a pathway, but they rarely display complete enzyme specificity. SU9516 and Fascaplysin are pan-CDK inhibitors with selectivity for CDK2 and CDK4 *in vitro* and *in vivo* [29], [30]. Because the effect on cJun phosphorylation was observed between 0 and 6 hours, we focused on CDK4 due to its relatively early activity in initiating cell proliferation, and tested the role of CDK4 in c-Jun phosphorylation using more direct approaches.

**Figure 2:**
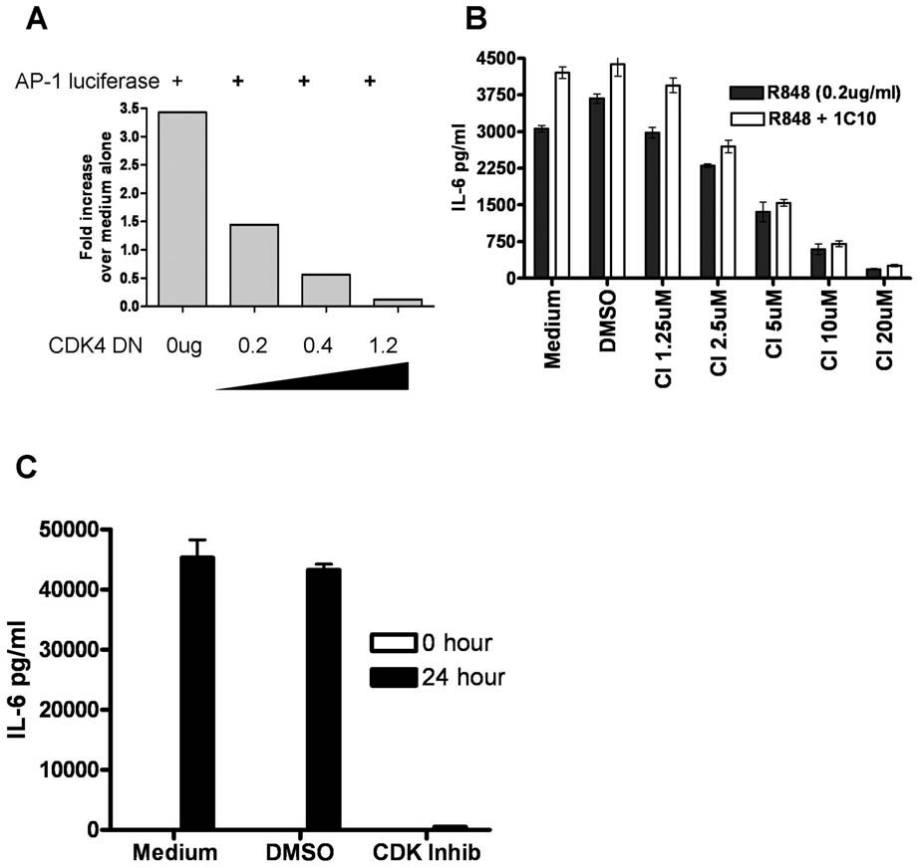
Effect of CDK inhibition on AP-1 function. A) 293T cells were transfected with increasing amounts of a plasmid encoding kinase dead CDK4, together with a construct producing murine TLR7 and an AP-1-luciferase reporter plasmid. Empty pRSV.neo plasmid was added to equalize the total amount of transfected DNA. After 24 hours of stimulation with the TLR7 agonist R848, relative amounts of luciferase activity were measured. B) IL-6 production by B cells stimulated with the agonistic anti-mouse CD40 antibody 1C10 and R848 with or without the CDK inhibitor SU9516 was quantified by IL-6-specific ELISA assay, as described in Methods. C) IL-6 production by purified human peripheral B cells stimulated with R848 for 24 hours with or without the CDK inhibitor SU9516 was monitored by ELISA. These data are each representatives of 3 separate experiments.

Using the easily transfected epithelial cell line 293T [31], we tested the capacity of a kinase-dead CDK4 mutant molecule (R158N) to inhibit AP-1 activity. Expression of the kinase dead, dominant negative CDK4 R159N mutant inhibited TLR-induced AP-1 reporter gene activity in a dose dependent manner (Fig. 2A). Because B cell production of IL-6 is dependent upon the activity of c-Jun -containing AP-1 [32], we tested the downstream effects of CDK inhibition first by monitoring IL-6 production as a biologically relevant effector function. While cell viability was unaffected, the production of IL-6 was dramatically reduced in mouse B cells treated with the CDK inhibitor SU9516 (Fig. 2B) and human B cells treated with the CDK4 specific inhibitor CINK (Fig. 2C).

### Effects of cellular proliferative status on CDK4 regulation of AP-1

**Figure 3:**
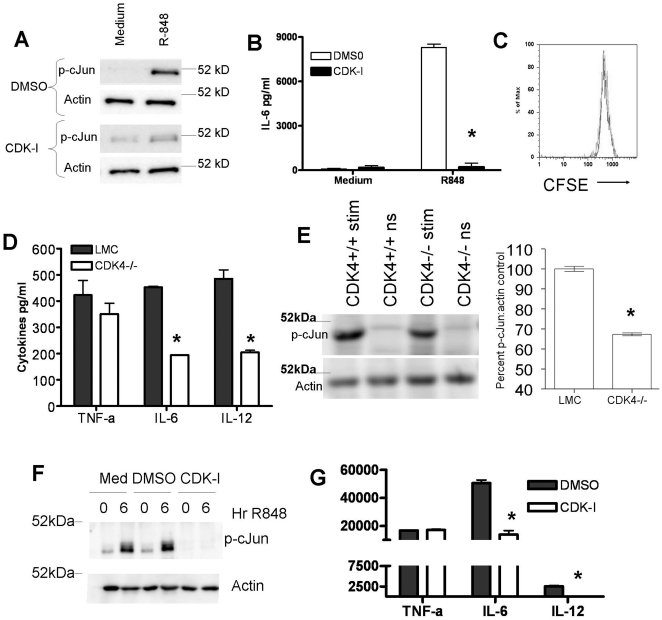
Effects of CDK4 inhibition on cytokine production and cJun phosphorylation in non-dividing cells. Differentiated BMDCs were stimulated with the TLR7 agonist R848 in the presence and absence of CDK-I (SU9516) for 6 and 24 hours and monitored for A) cJun phosphorylation and B) IL-6 production, as described in Methods. C) As indicated by CFSE staining, matured BMDCs did not proliferate upon TLR7 stimulation – DCs were either fixed (light grey line) or stimulated with R848 for 48 hours (dark grey line) D) BMDCs from CDK4 deficient mice and CDK4+/+ littermate controls were stimulated with R848 (1 ug/ml) for 24 hours and supernatants subjected to cytokine multiplex analysis. E) BMDCs from CDK4 deficient mice and CDK4+/+ were stimulated with R848 (1 ug/ml) for 6 hours and assayed for cJun phosphorylation. The level of c-Jun phosphorylation in the CDK4 sufficient cells, as determined by the p-c-Jun:actin ratio, was set to 100%. The level of p-c-Jun in CDK4 deficient cells was normalized to the 100%. F) Human monocyte-derived macrophages were stimulated with or without R848 for 6 hours in the presence of medium, DMSO (drug diluent), or the CDK4-specific inhibitor CINK. Cell lysates were then prepared and analyzed by Western blot for the presence of phospho-cJun. Actin was measured as a loading control. G) Human monocyte-derived macrophages were stimulated with or without R848 for 24 hours in the presence of DMSO, or the CDK4-specific inhibitor CINK. Supernatants were collected and analyzed for cytokines using multiplex technology. (*) indicates statistical significance – P-value <0.05 using Student t-test. These data are each representative of between 2 and 4 separate experiments.

The *in vitro* kinetics of cJun phosphorylation described above suggest that CDK-mediated c-Jun phosphorylation occurred prior to cell proliferation. To further explore the possibility that CDK4 regulates c-Jun phosphorylation independent of cellular proliferation, we examined the role of CDK4 in the ability of terminally differentiated bone marrow dendritic cells (BMDCs) to produce IL-6 and phosphorylate c-Jun (fully differentiated BMDCs did not undergo proliferation in response to TLR stimulation - Fig. 3). BMDCs stimulated through TLR7 and treated with CDK inhibitor showed a decrease in c-Jun phosphorylation and a concomitant decrease in IL-6 production, supporting the concept that CDKs are utilized in c-Jun/AP-1 signal transduction (Fig. 3A and B). To corroborate these findings, BMDCs were expanded from Wt and CDK4^−/−^ mice and tested for c-Jun phosphorylation and cytokine production upon TLR stimulation. Despite comparable *in vitro* differentiation and activation, as determined by CD11c and CD86 expression respectively (Fig. S1), CDK4^−/−^ BMDCs produced significantly less IL-6 and IL-12 compared to WT BMDCs from littermate controls. Interestingly, the level of TNF-α produced was not significantly different between the two groups, indicating that different cytokines may have different levels of dependence upon CDK-regulated c-Jun phosphorylation and AP-1 activity (Fig. 3C). In addition, the level of phosphorylated c-Jun was ∼30% lower in cells from the CDK4^−/−^ mice (Fig. 3D). Importantly, human myeloid cells displayed similar p- c-Jun and cytokine production deficiencies when treated with a CDK4 specific inhibitor (Fig. 3E and F).

**Figure 4:**
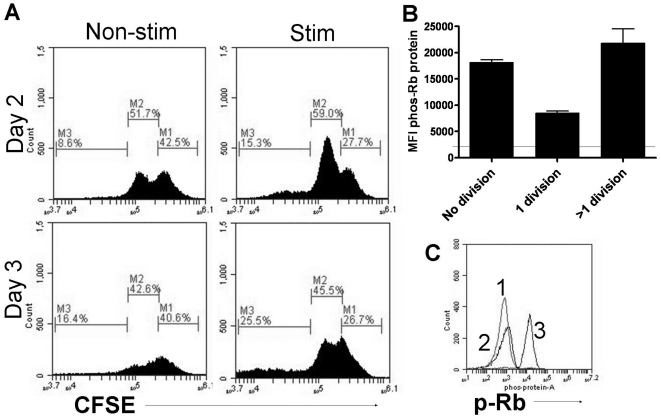
CDK4 activity in non-dividing cells. A) Mixed myeloid cells expanded from bone marrow of C57Bl/6 mice were stained with CFSE prior to stimulation with the TLR7 agonist R848. After 48 and 72 hours cells were fixed and stained for phosphoryated Rb protein on the CDK4 specific site Ser 780. Cells in gate M1 are CFSE high and represent non-dividing cells. The percentage of cells within this gate remained approximately 26% for three days. Cells in gates M2 and M3 are CFSE low and represent cells that have gone through 1 or more than 1 division respectively. From day 2 to day 3 the % of M2 cells has decreased while the percent of cells in M3 has increased indicating ongoing proliferation. B) A histogram of MFI for phospho-Rb from each of the M gates indicates phosphorylation of Rb protein in all stages of division (line represents MFI of non-stimulated cells). C) Histogram of cells from M1 gate stained for phospho-Rb protein after 48 hours of R848 stimulation. Peak 1 is the isotype control of stimulated cells, peak 2 is the p-Rb staining of non-stimulated cells, and peak 3 is the p-Rb staining of R848 stimulated cells. These data are representative of two individual experiments.

To further analyze the activity of CDK4 in non-dividing cells, the phosphorylation of the CDK4 specific target Ser 780 of Retinoblastoma protein was monitored in a mixed myeloid population expanded from bone marrow. Phosphorylated Rb was observed in both dividing (CFSE low) and non-dividing (CFSE high) populations using flow cytometry, thus demonstrating that CDK4 can be active in non-dividing cells (Fig. 4).

### Effects of TLR7 and CD40 stimulation on CDK4 activation

**Figure 5:**
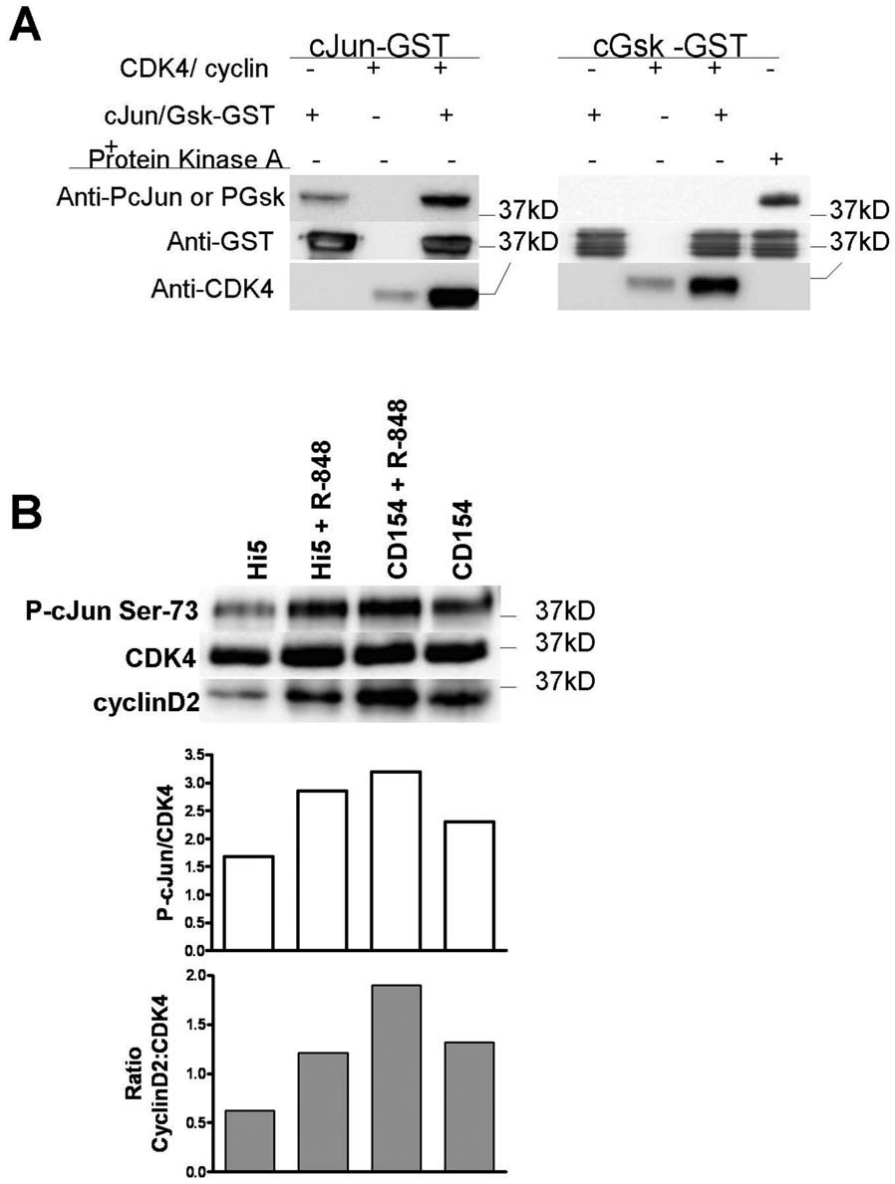
Phosphorylation of cJun by cellular CDK4. A) Purified recombinant CDK4:cyclinD complex was reacted with cJun and GSK GST fusion proteins to determine specificity of phosphorylation by CDK4. Western blotting with phospho-specific Abs was used to monitor the phosphorylation of the GST-fusion proteins. These data are representative of 3 separate experiments. B) CDK4 was immunoprecipitated from differentially stimulated B lymphocytes and reacted with GST-cJun fusion protein as described in Methods. The presence of cyclin D2 and phosphorylation of c-Jun was analyzed by Western Blot using anti-Ser 73 phospho-cJun Abs. Blotting against CDK4 was used as a loading control. Quantitative analysis of chemiluminescent band development enabled comparison of relative protein amounts between treatments (bar graphs). These data are representative of 2 separate experiments.

The CDK4 dependence of cJun phosphorylation could be explained by either direct interaction or indirect consequences. To test the possibility of a direct interaction between CDK4 and c-Jun, purified CDK4/cyclinD complex was tested for its ability to phosphorylate a c-Jun -GST fusion protein. The CDK4/cyclin D complex phosphorylated c-Jun but was unable to phosphorylate the control substrate Gsk-α, indicating that the direct c-Jun phosphorylation was substrate specific (Fig. 5A). To determine if this interaction occurs upon stimulation of cells, CDK4 was immunoprecipitated from differentially stimulated B lymphocytes and incubated with c-Jun -GST fusion proteins in an *in vitro* kinase assay. These CDK4 complexes phosphorylated c-Jun (Fig. 5B). These data, together with previous results suggest that CDK4 phosphorylates c-Jun *in vivo*. Consistent with early CDK4 activation events, the level of cyclin D2 association with CDK4 was increased upon B cell stimulation, and the level of associated cyclin D2 correlated with enhanced c-Jun phosphorylation (Fig. 5B).

## Discussion

The biological role of CDKs was previously thought to be limited to regulating cell proliferation. However, recent findings suggest that CDKs are key regulators of non-proliferative cellular functions such as cell mobility and survival [8], [9], [10], [11]. The molecular mechanisms by which CDKs mediate these functions are unknown. We show here that CDK4 directly phosphorylated c-Jun following mouse and human immune cell stimulation, leading to activation of the transcription factor AP-1 and enhanced production of AP-1 dependent cytokines. This is in direct support of a recent publication by Cho et. al. indicating that c-Jun is a target for CDK3 and therefore a regulator of AP-1 function necessary for proliferation and transformation [24]. In addition to this, data presented here using non-dividing BMDCs, indicate that CDK4 activity and AP-1 regulatory mechanism(s) are independent of cell proliferation.

In addition to identifying a new CDK4 substrate, these findings provide insights into the role of CDKs in non-proliferative cell functions, and highlight implications for the pharmacological effects of therapeutic CDK inhibition. Currently, CDK inhibitors are being tested as therapies for autoimmune disease and cancer [17], [33], [34]. Alvocidib, a CDK inhibitor and potential anti-cancer drug, abrogates experimental arthritis in mouse models [17]. The original explanation of Alvocidib's effect was blocking synovial fibroblast proliferation. While the drug does have this effect, there may be additional biological effects of CDK inhibitors in arthritis. Our results indicate that CDK inhibitors may also reduce the production of IL-6 and IL-1 by reducing the activity of AP-1. Previous studies report that neutralizing antibody therapy against IL-6 reduces arthritis scores and severity in both mice and humans [35], [36], [37], [38]. With regards to cancer immunotherapy, the blockade of IL-6 has been shown to reduce tumor growth in myeloma models [39]. Our data suggest that the use of CDK inhibitors may block production of IL-6 and therefore be of benefit to myeloma patients. However, the use of CDK inhibitors to treat some cancers may inhibit anti-tumor immune responses and therefore counter some of the effects of this drug treatment.

Our results focus on CDK4's ability to phosphorylate c-Jun, but other CDK family members may also regulate AP-1 through a similar mechanism to that demonstrated for CDK4. In addition to the partially redundant nature of CDKs, the common SAP kinase- phosphorylated residues of c-Jun (Ser63, 73 and Thr91 and 93) each contain a weak CDK consensus sequence (S/T – P) [40], indicating potential for other CDKs to phosphorylate c-Jun. This may be why Wt cells treated with pan-CDK inhibitors exhibited a more complete inhibition of c-Jun phosphorylation than did CDK4 deficient cells (Figs. 2 and 4). The ability of additional CDKs to phosphorylate cJun and regulate AP-1 is currently being investigated, and we are searching for other CDK targets. Intriguingly, other AP-1 subunits (JunD, B and cFos) contain weak CDK consensus sequences and therefore may be phosphorylated/regulated by CDKs, thus further expanding the known functions of CDKs.

Our findings indicate that CDK4 can regulate the function of AP-1 through the direct phosphorylation of c-Jun, and therefore potentially regulates a number of cellular functions independent of replication. This newly described molecular interaction of a CDK may have important implications for treatment of autoimmune and inflammatory diseases, as well as suggesting new properties relevant to the use of CDK inhibitors as anti-cancer reagents.

## Materials and Methods

### Reagents

CDK inhibitors (SU9516, Fascaplysin, and CDK4 inhibitor - CINK) and JNK inhibitor VIII were purchased from Calbiochem (San Diego, CA). The construct encoding constitutively active human CDK4 (CDK4-R24C) was subcloned from the pBABE-CDK4-R24C construct (a generous gift from Dr. A. Kingelhutz, The University of Iowa, Iowa City, IA) into the popRSV.neo vector [41]. This popRSV - CDK4-R24C construct was used as the template for production of the CDK4 kinase dead mutant, made by altering aspartic acid 158 to asparagine using a point mutagenesis kit from Stratagene (Cedar Creek, TX) [42].

Antibodies (Abs) used for Western blots, intracellular cytokine staining and immunoprecipitations were as follows: anti phospho- c-Jun Ser73 Abs were purchased from either Upstate Biotechnology (Lake Placid, NY) or Biosource (Carlsbad, CA). The anti-phospho p38, total p38, phospho-JNK, total ERK 1/2, phospho-Retinoblastoma protein (Ser 780), and GST Abs were purchased from Cell Signaling Technologies (Beverly, MA). Abs against phospho-ERK 1/2 were purchased from Biosource and the Abs against total JNK, CDK4, cyclin D2, cyclin D1, CDK6 and CDK2 were purchased from Santa Cruz Biotechnology (Santa Cruz, CA). Antibodies used for flow cytometric analysis of DCs (anti-CD11c and CD86) were purchased from eBioscience (San Diego, CA). Anti-mouse IL-6 mAbs (MP5-20F3 and MP5-32C11, biotin-conjugated) used for ELISA were also obtained from eBioscience. Multiplex cytokine assay (mouse 20-plex) was purchased from Biosource and used according to manufacturer's instructions.

Enzymatically active, purified CDK complexes were purchased from Calbiochem. Fusion proteins c-Jun (residues 1–79)-GST and GSK-3α-GST were purchased from Upstate Biotechnology and BioVision (Mountain View, CA) respectively. The anti-mCD40 agonistic antibody 1C10 was purified from a hybridoma kindly provided by Dr. Frances Lund (University of Rochester School of Medicine, Rochester, NY). The stimulatory TLR7 ligand R848 was obtained from Alexis Biochemicals (San Diego, CA). CD154-expressing Hi5 insect cells have previously been described [16].

### Cells

Hi5 insect cells expressing CD154 have been previously described [43]. Primary small dense (resting) splenic B lymphocytes were harvested from C57Bl/6 mice by Percoll gradient centrifugation as previously described [44]. Purity was monitored by flow cytometry with anti-CD19 mAb (eBioscience). Purity of cells used was >92%. Human peripheral B cells were isolated using a negative isolation kit (StemCell Technologies, Vancouver, British Columbia) according to manufacturer's instructions. Purity of cells was >90%. Stimulation of cells through CD40 used either Hi5 insect cells expressing the CD40 ligand (CD154) at a ratio of 1:5 Hi5 to immune cell, or using anti-CD40 monoclonal Ab, 1C10. Bone marrow derived dendritic cells (BMDCs) were expanded and matured as previously described [45]. Unlike B cells, BMDCs stimulated via CD40 did not respond with easily detectable cJun phosphorylation or IL-6 production, so for these cells, TLR stimulation alone was used. Bone marrow samples from CDK4 deficient mice and littermate controls were kind gifts from Dr. L. Schnapp, University of Washington, Seattle, WA and Dr. H. Kiyokawa, University of Illinois College of Medicine, Chicago, IL. Human myeloid cells were isolated and expanded from peripheral blood (obtained from the DeGowin Blood Center at The U of Iowa) as previously described [46]. Mice were used in accordance with VAMC Animal Use guidelines.

### Western blots

Cells lysates were prepared as previously described [16] and separated using 10% SDS-polyacrylamide gels. Proteins were then transferred onto PVDF membranes, and analyzed by Western blot as previously described [16]. Peroxidase labeled secondary Abs were visualized using the chemiluminescent detection reagent West Pico peroxidase substrate (Pierce, Rockford, IL) and the luminescence measured using a Fujifilm LAS-1000 imaging system (Fujifilm Medical Systems, Ltd., Stanford, CT). Chemiluminescence was subsequently quantified using ImageGauge software (FujiFilm).

### IL-6 ELISA

Cells were treated as described with R848, CD154-expressing or Wt Hi5 insect cells and/or chemical inhibitors (JNK VIII, and/or CDK inhibitors). Chemical inhibitors were added 30 minutes prior to stimulation and used at concentrations that did not decrease cell viability, as monitored by detection of subdiploid DNA using propidium iodide staining and flow cytometric analysis [47]. Cell free supernatants were collected and quantitative ELISA for IL-6 (eBioscience) was performed according to the manufacturer's recommended protocol.

### Luciferase assay

293T cells at 80% confluence were transfected in 2 ml cultures of a 6 well plate with increasing amounts of a plasmid encoding kinase dead CDK4, together with murine TLR7 (0.5 ug of plasmid) and an AP-1 driven luciferase reporter plasmid. The transfection utilized the reagent Lipofectamine (Invitrogen, Carlsbad, CA) in accordance with manufacturer's instructions. Empty pRSV.neo vector plasmid was added to equalize the total amount of transfected DNA. 24 hours after transfection, cells were harvested from the 6 well plate, washed in growth medium, and plated at 1×10^5^ cells per well in a 48 well plate. Cells were then treated with medium or the TLR7 agonist R848 (1 ug/ml) for 20 hours and the relative amounts of luciferase activity were measured using a dual reporter kit according to manufacturer's instructions (Promega, Madison, WI).

### Proliferation measurements and intracellular staining for phospho-Rb

Fully differentiated BMDCs were expanded as described [45] and stained with CFSE (Molecular Probes – Eugene, Oregon) as per the manufacturer's instruction. Upon mitogenic stimulation, the decrease in CFSE as it is portioned into daughter cells is indicative of cellular division. Cells were stimulated as described and monitored for division by flow cytometry. Partially differentiated myeloid cells were expanded by culturing bone marrow cells in GMCSF and IL-4 as described for BMDCs for 48 hours. After 48 hours, cells were stained with CFSE and stimulated with R848 for 48 and 72 hours. Cells were then fixed and permiablized using methanol as previously described [48]. Cells were then stained using an antibody against pRb protein or isotype control antibodies (Cell Signaling Technologies) followed by a goat anti-rabbit antibody labeled with APC –fluorochrome (Molecular Probes). Cells that retained the same level of CFSE over the course of three days were considered non-dividing. Flow cytometry was performed on an ACCURI cytometer.

### Immunoprecipitations (IP) and kinase assays

Freshly isolated resting B cells (2×10^6^ cells) were stimulated with R848 (1 ug/ml) and CD154-expressing Hi5 insect cells or Hi5 cells without CD154 expression for 5 hours. Cells were then pelleted by centrifugation (2 minutes at 500 x g) and resuspended in 0.5 ml lysis buffer (50 mM HEPES, pH 7.3, 150 mM NaCl, 1 mM EDTA, 2.5 mM EGTA, 10 mM beta-glycerophosphate, 0.1 mM sodium orthovanadate, 0.1% Tween-20, and 10% glycerol, with 0.1 mM PMSF, 1 mM DTT 1 mM NaF added just before use). Cells were incubated at 4°C for 30 minutes. Anti-CDK4 Ab (10 ug/ml) and 20 ul of protein A-Sepharose were added to the cell lysates, which were rotated at 4°C for 2 hours. IP complexes were washed 4 times in lysis buffer and 2 times in kinase buffer (50 mM HEPES, pH 8.0, 10 mM MgCl_2_). The complexes were then resuspended in 30 ul kinase buffer. 1 ul of 100 mM ATP and 0.5 ug c-Jun -GST fusion protein were added to IP suspensions. Kinase assays using purified CDK complexes were performed using a reaction mixture of 0.2 ug kinase complex or PKA, 1 ul of 100 mM ATP, and 0.5 ug GST fusion protein diluted to a final volume of 35 ul with kinase buffer. Kinase reactions proceeded at 30°C for 20 minutes with shaking (1000 rpm on a heated shaker block). Reactions were stopped by the addition of 12 ul of 4X SDS treatment buffer. The samples were heated to 95°C for 10 minutes and subjected to Western blotting.

## Supporting Information

Fig. S1 **Dendritic cells from CDK4 deficient mice show mature phenotype.** BMDCs were isolated and expanded as described in the material and methods. Cells were then stimulated with R-848 (1 ug/ml) for 24 hours and stained for expression of A) CD11c, a DC marker and B) CD86, an activation marker.
(TIF)
