# Supplementary figures and images for: CDK-Mediated Regulation of Cell Functions via c-Jun Phosphorylation and AP-1 Activation
Source: PLoS One. 2011 Apr 29;6(4):e19468. doi: 10.1371/journal.pone.0019468 (PMC3084876; doi:10.1371/journal.pone.0019468)

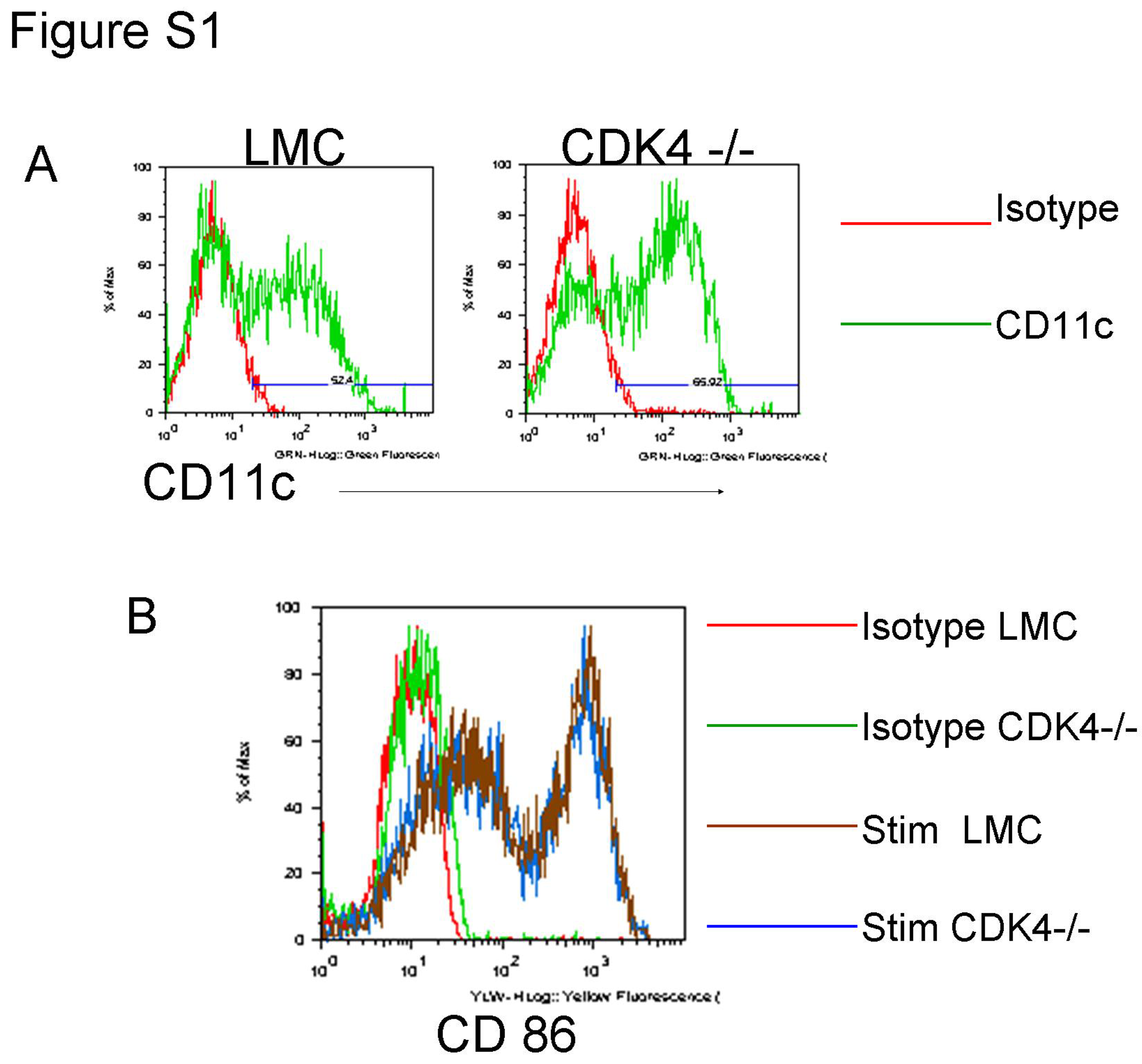

Supplement: Fig. S1 — Dendritic cells from CDK4 deficient mice show mature phenotype. BMDCs were isolated and expanded as described in the material and methods. Cells were then stimulated with R-848 (1 ug/ml) for 24 hours and stained for expression of A) CD11c, a DC marker and B) CD86, an activation marker. (TIF) [file pone.0019468.s001.tif]
